# Astragaloside IV Protects Ethanol-Induced Gastric Mucosal Injury by Preventing Mitochondrial Oxidative Stress and the Activation of Mitochondrial Pathway Apoptosis in Rats

**DOI:** 10.3389/fphar.2019.00894

**Published:** 2019-08-15

**Authors:** Shumin Qin, Jinjin Yin, Shaogang Huang, Jingyu Lin, Zhigang Fang, Yunsong Zhou, Keer Huang

**Affiliations:** ^1^Department of Gastroenterology, The Second Affiliated Hospital of Guangzhou University of Chinese Medicine, Guangzhou, China; ^2^Department of Pharmacy, The Third Affiliated Hospital of Guangzhou Medical University, Guangzhou, China; ^3^Fujian Provincial Institute of Traditional Chinese Medicin, Fuzhou, China; ^4^The First Affiliated Hospital of Guangzhou University of Chinese Medicine, Guangzhou, China

**Keywords:** astragaloside IV, mitochondrion, oxidative stress, apoptosis, ethanol

## Abstract

Alcohol consumption affects gastric mucosa by multiple and complex mechanisms depending either by direct contact of ethanol or by indirect biological damage induced by its metabolite acetaldehyde. The present study aims at further investigating the mechanism of ethanol-induced gastric mucosa injury and the protective effect of astragaloside IV (AS-IV) in an aspect of mitochondrial oxidative stress and mitochondrial pathway of apoptosis. Using an array of experimental approaches, we have shown that the development of mitochondrial oxidative stress and associated apoptosis play crucial roles in the pathogenesis of gastric injury induced by ethanol. AS-IV inhibits mitochondrial oxidative stress by scavenging accumulation of malondialdehyde and decreasing the consumption of glutathione. AS-IV also prevents ethanol-induced apoptosis by modulating the activity of caspase-3 and caspase-9, the expression of Bax/Bcl-2, and the release of cytochrome C and apoptosis inducing factor. Moreover, AS-IV reduces ethanol-mediated activation of caspase-8 and breakage of Bid. This study thus indicates that AS-IV prevented ethanol-induced gastric damage by blocking activation of mitochondrial oxidative stress and mitochondrial pathway of apoptosis induced by ethanol in the gastric mucosa.

## Introduction

As one of the socially accepted addictive drugs worldwide over the period of time, alcohol consumption has been linked to various diseases (Piazza-Gardner et al., 2013; Ceni et al., 2014; Rocco et al., 2014; Knott et al., 2015; Liew et al., 2016). The incidence of gastric mucosal lesions including gastritis, gastric ulcer, and even gastric carcinoma in drinking population is significantly higher than that in nondrinking populations (Akiyama et al., 2008; Anderson et al., 2009; Minami et al., 2018; Wang et al., 2018; Yu et al., 2018). As an important causative factor, ethanol induces gastric mucosal injury through multiple and complex mechanisms depending either by direct contact of itself or indirect biological damage induced by its metabolite acetaldehyde. The direct effects include disruption of mucosal cellular membranes, dehydration, and cytotoxic effect with consequent propagation of the inflammatory cascade (Park et al., 2008). Meanwhile, due to lack of acetaldehyde dehydrogenase (ALDH), accumulation of acetaldehyde metabolized from 10% of alcohol in the stomach induce indirect damage of the gastric mucosal cells by triggering inflammatory responses and oxidative stress, which could give rise to cellular apoptosis by both the mitochondria-dependent and mitochondria-independent pathway (Arab et al., 2015). As a kind of important cellular organelle, mitochondrion is the main site for the production of energy and reactive oxygen species (ROS), which plays a crucial role in metabolism as well as maintenance of homeostasis in cell. Augmentation of ROS generation leads to injury of mitochondrial lipids, proteins, and nucleic acids and thereby disrupts mitochondrial function (Murphy and Smith, 2007). Studies showed that the mitochondria-pathway apoptosis is involved in the pathogenesis of gastric damage induced by nonsteroid anti-inflammatory drugs (NSAIDs), *Helicobacter pylori*, and stress (Calvino-Fernandez et al., 2008; Maity et al., 2009; Pal et al., 2010; De et al., 2017). Moreover, oxidative metabolism of ethanol sensitizes pancreatic mitochondria to activate MPTP leads to mitochondrial failure (Shalbueva et al., 2013). This research was to study whether development of mitochondrial oxidative stress and associated dysfunction play crucial roles in the pathogenesis of gastric injury induced by ethanol as which was not definitively established.

As a kind of cycloartane-type triterpene glycoside, Astragaloside IV (AS-IV) is extracted from the dried root of leguminous plants Milkvetch Root [*Astragalus membranaceus* (Fisch.) Bunge.], which has been used for thousands of years to treat digestive diseases, and presents multiple biological functions including antioxidant, antiasthma, antidiabetes, protection of cardiovascular, and improvement of immune function (Luo et al., 2004; Jin et al., 2010; Li et al., 2013; Shao et al., 2014). The gastroprotective action of AS-IV has been established in our previous study (Qin et al., 2017). It was reported that AS-IV protected tubular damage, acute stroke, and myocardial injury by exerting its effects of antioxidative stress and anti-apoptosis (Qi et al., 2014; Xu et al., 2018; Yin et al., 2018). Furthermore, recent literatures showed that AS-IV prevented aging and Parkinson’s through effect of antioxidant and regulation of mitochondrial function (Liu et al., 2017), as well as inhibited oxidative stress-induced mitochondrial permeability transition pore opening in H9c2 cardiac cells (He et al., 2012). It is indicated that AS-IV possesses the potential of modulating mitochondrial homeostasis and preventing apoptosis. However, further research is required to ascertain whether AS-IV exhibits the similar effect on protecting ethanol-induced gastric mucosal injury. Therefore, the involvement of mitochondrial oxidative stress and related apoptosis in the development of ethanol-induced gastric injury and the effect of AS-IV on this process in a rat model of ethanol-induced gastric mucosal injury were detected. The present study showed that ethanol induced significant mitochondrial oxidative stress in gastric mucosal cells *in vivo* characterized by accumulation of lipid peroxide and consumption of glutathione (GSH) and manganese-based superoxide dismutase (Mn-SOD) in mitochondria. The mitochondrial oxidative stress caused activation of mitochondrial-dependent apoptotic pathway, which led to mucosa injury. AS-IV attenuated the ethanol-induced mitochondrial oxidative stress by scavenging lipid peroxide and prevented gastric mucosal cells apoptosis through inhibition of the mitochondrial pathway.

## Materials and Methods

### Materials

AS-IV (>99%) was purchased from the National Institutes for Food and Drug Control, China. The mitochondrial isolation kit, caspase-3, caspase-9, and caspase-8 Colorimetric Assay Kits were obtained from Biovision, USA. The detection kits of GSH and Mn-SOD were obtained from Jiancheng Bioengineering Institute, China. TUNEL assay kit was from KeyGEN Biotech Co. Ltd, China. Protease inhibitors (complete mini tablets) were purchased from Roche, Germany. Antibodies of Bax, Bcl-2, and Bid were from Abcam, Novus, Millipore, USA. The detection kit of MDA and antibodies of AIF and Cyt-c were purchased from Beyotime Institute of Biotechnology, China. The mitochondrial membrane potential fluorescence detection kit was obtained from GENMED, China. Ultrapure RNA extraction kit, Reverse Transcription System, and miRNA Real-Time PCR assay kit were from TaKaRa, Japan.

### Animals and Ethanol-Induced Gastric Mucosal Damage

Adult male Sprague–Dawley rats weighing 180–220 g were used in this study [animal permit number: SCXK (Yue) 2013-0056]. The rats were maintained at controlled laboratory conditions [temperature (25 ± 1°C), humidity (60 ± 10%), and a 12/12-h light/dark cycle]. Animals were allowed 1 week for acclimatization before any experimental procedures, and they had free access to standard rat chow and water. Gastric mucosal injury was induced using a single intragastric dose of absolute alcohol (5 ml/kg) that was administered via orogastric intubation. The control group received the same volume of saline instead of ethanol. Rats were placed individually in metabolic cages with raised floors of wide mesh to avoid coprophagia that interfere with the induction of gastric injury.

### Preparation of AS-IV Injection

The preparation of AS-IV injection was conducted as described (Qin et al., 2017). Briefly, appropriate amounts of AS-IV were dissolved in solvent blended anhydrous ethanol and propylene glycol in the ratio of 2:1 (invention patent, China, 200510014971.2), sonicated 30 min to plane solubilis, then diluted with sterilizing water for injection to10%.

### Experimental Design and Treatment Protocol

Animal grouping and intervention were conducted as described (Qin et al., 2017). In brief, after 1 week for acclimatization, animals were randomly divided into four experimental groups with eight rats in each group. The control and model groups received daily i.p. injections of saline at 1 ml/100 g for 4 days. The animals in the AS-IV group received daily i.p. injections of AS-IV at 4 mg/kg for 4 days (final volume: 1 ml/100 g). The rats in the solvent group were injected 10% solvent blended anhydrous ethanol and propylene glycol in the ratio of 2:1 at 1 ml/100 g for 4 days. On the third day of the experiment, all the animals were fasted for 24 h but had free access to water. On the fourth day, the rats were water deprived after receiving intraperitoneal injection; 75 min later, absolute ethanol was given orally to animals except the control group at 5 ml/kg; the control group received the same volume of saline orally. After 1 h of ethanol treatment, rats were euthanatized, and the stomachs were collected.

### Determination of Macroscopic and Histopathological Injury of Gastric Mucosa

Macroscopic and histopathological examinations were managed as in our previous study (Qin et al., 2017). Briefly, an electronic vernier caliper was used to measure the length, width, and diameter of lesions in flattened stomach samples, and injury score was calculated to evaluate the macroscopic damage. Injury scores = ∑(A) + (2B) + (C) [A is the length (mm) of linear lesion, with width up to 1 mm; B is the length of linear lesion with width >1 mm; C is the area (mm^2^) of irregular and rounded lesion]. For pathological assessment, a specimen of each stomach was fixed in 10% formalin solution and then embedded in paraffin. Embedded sections were cut using the microtome at a thickness of 4 μm and stained with hematoxylin and eosin (H&E) examined under a light microscope (Leica Microsystems, Germany), Gastric microscopic damage was scored on a 0–14 scale according to the criteria previously reported (Laine and Weinstein, 1988). Briefly, a 1-cm segment of each histological section was examined for: (1) mucosal edema (score 0–4), (2) hemorrhage (score 0–4), (3) inflammatory cell infiltration (score 0–3), and (4) epithelial cell loss (score 0–3).

### Assay of Apoptosis in Gastric Mucosal Tissue

The apoptosis in gastric mucosal tissue was evaluated by detection of DNA damage using the TUNEL Assay Kit. The explanation of operation is in accordance with the instruction. In brief, gastric mucosal tissues obtained from the same position of stomach of rats in each group were fixed in 10% formalin solution, embedded in paraffin, and then cut at a thickness of 4 μm. The paraffin sections were dewaxed using ethanol and xylene. After being washed and permeated, the samples were loaded with 100 μl of TdT enzyme reaction solution and humidified at 37°C for 1 h. After being washed three times with PBS, each sample was added with 100 μl of Streptavidin-HRP and humidified at 37°C for 30 min. Then, freshly prepared DAB solution was added and the chromogenic reaction was stop using distilled water. It was counterstained in hematoxylin for 10 min and rinsed with distilled water. Finally, after covering the slide, the samples were observed under a light microscope (Leica Microsystems, Germany). The numbers of apoptotic cells were counted in each of the five fields under 400× multiples. The apoptosis rate was calculated and recorded.

### Isolation and Identification of Mitochondria

The extraction and purification of mitochondrial were administrated using a mitochondrial isolation kit (Biovision, USA) following the manufacturer’s instructions. In brief, after being washed several times and minced in ice-cold mitochondria isolation buffer, the gastric mucosal scrap from each group were collected and homogenized using Ultra-Turax T10 homogenizer (IKA, USA). The homogenate was transferred to a tube and centrifuged at 600×*g* for 10 min at 4°C. The supernatant was collected in a separate tube and centrifuge at 7,000×*g* for 10 min at 4°C. The supernatant was collected as cytosolic fraction. The mitochondria pellet was resuspended in storage buffer and identified with 0.5% Jenas Green B dye.

### Mitochondrial Oxidative Stress Measurement

To study whether mitochondrial oxidative stress plays a pathogenic role in the development of ethanol-induced gastric damage, the status of mitochondrial oxidative stress was measured in the mitochondria of gastric mucosal cell in terms of lipid peroxidation, GSH depletion, and Mn-SOD activity. The mitochondrial fraction was sonicated in 20 mm ice-cold ethylenediaminetetraacetic acid (EDTA) to detect the levels of MDA and GSH, as well as the activity of Mn-SOD. First, the mitochondrial protein content was detected by a Micro BCA protein assay kit. As the product of lipid peroxides, the mitochondrial MDA was measured referring to the instructions of Lipid Peroxidation MDA Assay Kit (Beyotime, China). The mitochondrial lysates were reacted with the thiobarbituric acid (TBA), and the reaction products were measured spectrophotometrically at 535 nm. The experiment was repeated three times, and the MDA levels were expressed as μmol/mg protein. The Mn-SOD assay kit and GSH assay kit (Jiancheng Bioengineering Institute, China) were selected for Mn-SOD and GSH measurement, and the assays were conducted according to the manufacturer’s instruction.

### Mitochondrial Transmembrane Potential

Mitochondrial transmembrane potential was measured using a mitochondrial membrane potential fluorescence detection kit and according to the manufacturer’s protocol (GENMED, China). In brief, mitochondria were isolated from gastric tissues using a mitochondrial isolation kit (Biovision, USA) following the method described above. Isolated mitochondria (25 µg) in 100 µL of JC-1 assay buffer (100 mM MOPS, pH 7.5, containing 550 mM KCl, 50 mM ATP, 50 mM MgCl_2_, 50 Mm sodium succinate, 5 mM EGTA) were incubated in the dark with JC-1 (300 nM) for 10 min at 25°C. The fluorescence of each sample was measured using a fluorescence microplate reader (excitation, 490 nm; emission, 530 nm). The relative fluorescence units (RFU) were measured and recorded to evaluate the mitochondrial transmembrane potential.

### Assay for Cytochrome c, AIF and Bid

Cytochrome c, AIF, and Bid in the cytosol were measured using Western blot. In brief, the cytosolic fraction from each group extracted as above was used to measure the expression of protein of cytochrome c, Bid, and AIF. Equal amounts of protein (60 μg) were subjected to 10% sodium dodecyl sulfate–polyacrylamide gel electrophoresis (SDS-PAGE) and blotted into a nitrocellulose membrane. The membranes were immunoblotted to detect cytochrome c, Bid, and AIF protein using rabbit anti-cytochrome c antibody (1:200), anti-Bid antibody (1:2,000), and anti-AIF antibody (1:200). The intensity of the bands in the membrane was measured using Image J.

### Assay of Caspase-3, Caspase-9, and Caspase-8

The activity of caspase-3, caspase-9, and caspase-8 was measured in the cytosolic fraction of the gastric mucosal homogenate using commercially available kits and according to the manufacturers’ protocol (Biovision, USA). In brief, gastric tissue was homogenized in caspase lysis buffer provided with the kit. After being incubated on ice for 10 min, the homogenate was centrifuged at 10,000×*g* for 1 min to a get clear supernatant. Assayed protein concentration and diluted 150 µg protein to 50 µl lysis buffer. A sample of 50 µl of supernatant was mixed with 50 µl of 2× reaction buffer provided with the kit in the presence of substrate (LEHD-ρNA for caspase-9, IETD-ρNA for caspase-8, and DEVD-ρNA for caspase-3, 200 µm final concentration). The mixture was incubated at 37°C for 1.5 h, and OD was taken at 405 nm.

### Western Blot for Bax and Bcl-2

Mitochondrial translocation of Bax and Bcl-2 was studied using Western blot. Mitochondrial pellets from each group were solubilized with radio immunoprecipitation assay (RIPA) lysis buffer containing protease inhibitors. Equal amounts of mitochondrial protein (30 μg), which was detected using a Micro BCA protein assay kit, were subjected to 10% sodium dodecyl sulfate–polyacrylamide gel electrophoresis (SDS-PAGE) and blotted into a nitrocellulose membrane. The membrane was immunoblotted for the detection of Bax and Bcl-2 protein using rabbit anti-Bax antibody (1:2,000) and anti-Bcl-2 antibody (1:2,000). The intensity of the bands in the membrane was measured using Image J.

### RT-qPCR for Mitochondria-Associated Apoptosis-Related Factors

Reverse transcription-quantitative polymerase chain reaction (RT-qPCR) was used to assay the mRNA levels of Bax, Bcl-2, AIF, and Cyt-c. Equal amounts of gastric tissues (20 mg) from each group were used for total RNA isolation using a commercially available kit (TaKaRa). RNA purity and concentration were identified using a Nanodrop2000 ultrafine spectrophotometer. The cDNA was prepared by reverse transcription system (TaKaRa). Equal amounts of cDNA were used for PCR amplification using specific forward and reverse primers of Cyt c, AIF, Bcl-2, Bax, Bid, and GAPDH (**Table 1**). The qPCR was performed using SYBR Premix Ex Taq (TaKaRa) following the protocol: 95°C for 30 s for initial denaturation, then 40 cycles of denaturation at 95°C for 5 s, and annealing at 60°C for 34 s. The relative mRNA levels were normalized to GAPDH and quantified using the 2−^∆∆CT^ method.

**Table 1:** The primers of mitochondria-associated apoptosis-related factors for qPCR.

Apoptosis-related factors	Primer (5′ to 3′)
Bid-F	CAGTCCAGTCCTCCTTGA
Bid-R	ATGTTAGCGTGCCTTCTC
AIF-F	ATGCTGCTGCTACTACTAC
AIF-R	AAGACAGGACAGACTACATAC
Cyt-C-F	GCCTGGTGGTAGTATTGTAA
Cyt-C-R	CCTGTGCTCTGTCTGAAC
Bax-F	TGAAGGTGGAAGTAGAAGGA
Bax-R	ACAGCAGCACTCACGATA
Bcl-2-F	TTCTTGCCTTCTTGGACTC
Bcl-2-R	TGCTGGATGATGGATGATG
GAPDH-F	TCTATGGTATGGTGGTGACT
GAPDH-R	TCTCTTGCTCTCAGTATCCT

### Statistical Analysis

Data are presented as means ± standard error of the mean (SEM). Results were analyzed statistically by one-way analysis of variance (ANOVA) with subsequent multiple comparisons using Tukey–Kramer test. Nonparametric values were presented as median, and the statistical differences among groups were calculated using Kruskal–Wallis analysis of variance followed by the rank-based Mann–Whitney *U*-test. All statistical tests were performed using SPSS program, version 15. Differences were considered significant at *P* < 0.05.

## Results

### Macroscopic and Histological Examination

**Figure 1:**
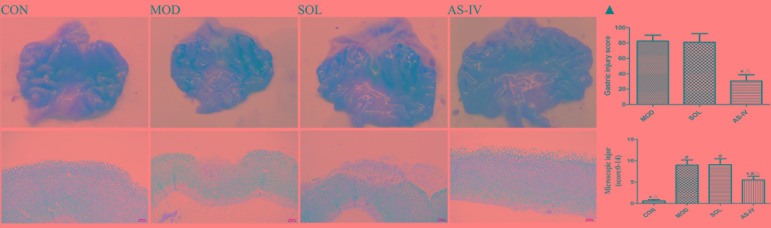
Intragastric administration of absolute ethanol triggered severe linear hemorrhagic damage and multifocal erosions in gastric mucosa, accompanied by submucosal edema, inflammatory cell infiltration, and epithelial cell loss (200×). Pretreatment with AS-IV alleviated these injuries. ▲ The analyses of gastric injury score and microscopic damage score. **CON**: control; **MOD**: model; **SOL**: solvent; **AS-IV**: AS-IV (4 mg/kg). Significant difference (*P < 0.05) when compared with the model group. Significant difference (#P < 0.05) when compared with the control group. Significant difference (∆P < 0.05) when compared with the solvent group. N = 6–8.

Intragastric administration of absolute ethanol triggered several linear hemorrhagic damage and multifocal erosions with high scores of microscopic damage characterized by hemorrhagic injury, submucosal edema, inflammatory cell infiltration, and epithelial cell loss compared to the vehicle-treated control group. As shown in **Figure 1**▲, pretreatment with 4 mg/kg of AS-IV significantly reduced gastric injury scores and pathological scores (*P* < 0.05). There was no difference between the solvent group and the model group in macroscopic damage and injury scores. These suggested that AS-IV attenuated the development of ethanol-induced gastric injury.

### The Effect of AS-IV on Apoptosis in Ethanol-Induced Gastric Injury

**Figure 2:**
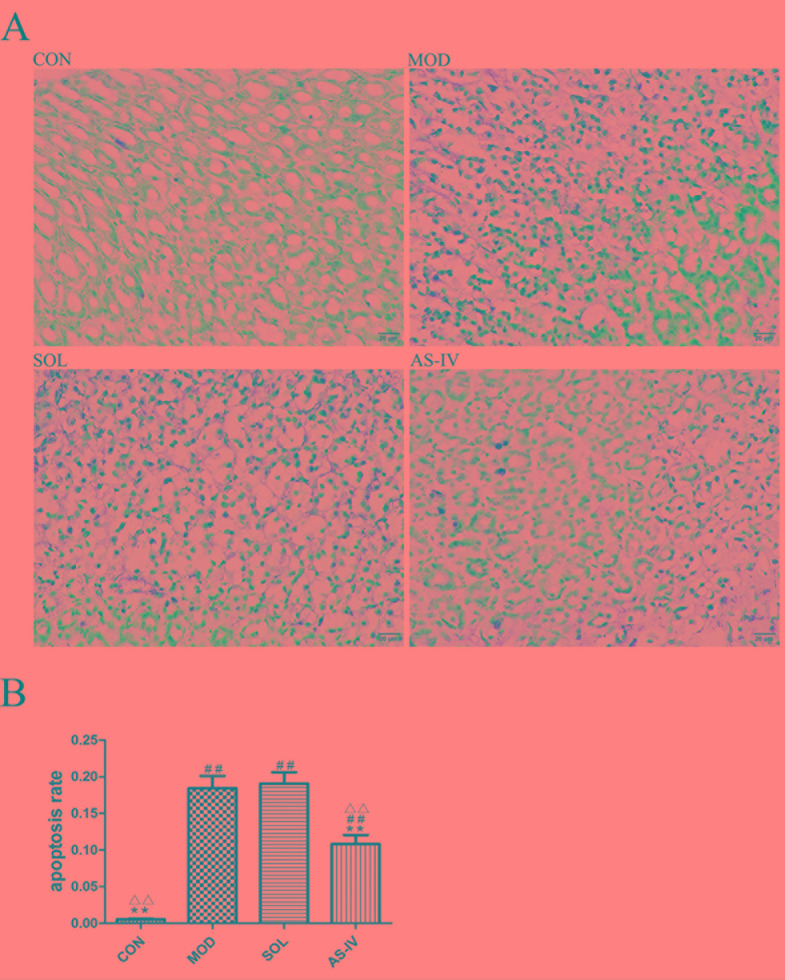
TUNLE assay of gastric mucosa tissue observed under a light microscope (400×). **(A)** Pretreatment with AS-IV reduced the dark brown staining, which is sign of apoptosis induced by ethanol; **(B)** The analysis of apoptosis rate. Significant difference (**P < 0.01) when compared with the model group. Significant difference (##P < 0.01) when compared with the control group. Significant difference (∆∆P < 0.01) when compared with the solvent group.

After TUNEL staining of paraffin sections of the gastric tissue, the DNA ruptured in the apoptotic cell nucleus specifically bound to HRP, and in the presence of DAB, a dark brown granular substance was produced. As shown in **Figure 2A**, the ethanol induced remarkable apoptosis of gastric mucosal cells, whereas pretreatment with AS-IV effectively inhibited apoptosis induced by ethanol. Meanwhile, the dark brown particles and normal cells were counted, and the apoptosis rate was calculated. The results showed that the apoptosis rate of AS-IV group was significantly lower than that of the model and solvent groups (*P* < 0.01). No difference was found between the model and solvent groups (**Figure 2B**).

### The Effect of AS-IV on Mitochondrial Oxidative Stress

**Figure 3:**
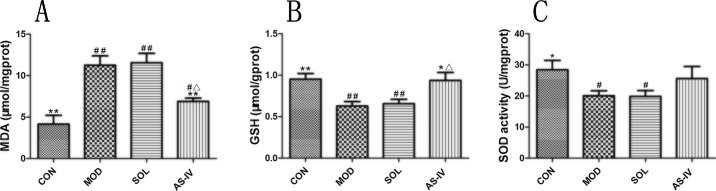
Ethanol-induced mitochondrial oxidative stress as revealed by reduction in mitochondrial GSH, Mn-SOD, and augmentation of MDA. Pretreatment with AS-IV prevented the changes of MDA and GSH. **(A)** Analysis of MDA level of mitochondrial fraction; **(B)** Analysis of GSH level of mitochondrial fraction; **(C)** Analysis of SOD activity of mitochondrial fraction. Significant difference (**P < 0.01, *P < 0.05) when compared with the model group. Significant difference (##P < 0.01, #P < 0.05) when compared with the control group. Significant difference (∆P < 0.05) when compared with the solvent group.

Ethanol induced mitochondrial oxidative stress in gastric mucosal cells as revealed by 34% reduction of GSH and 29% of Mn-SOD compared with control (*P* < 0.01 or *P* < 0.05), along with augmentation of MDA (more than 60% above control, *P* < 0.01). Pretreatment with AS-IV significantly attenuated the mitochondrial MDA and suppressed the decrease in GSH. Despite the increasing trend of Mn-SOD activity in the AS-IV group, the difference between model and AS-IV groups was not significant (*P* > 0.05) (**Figure 3**).

### The Effect of AS-IV on Mitochondrial Transmembrane Potential

**Figure 4:**
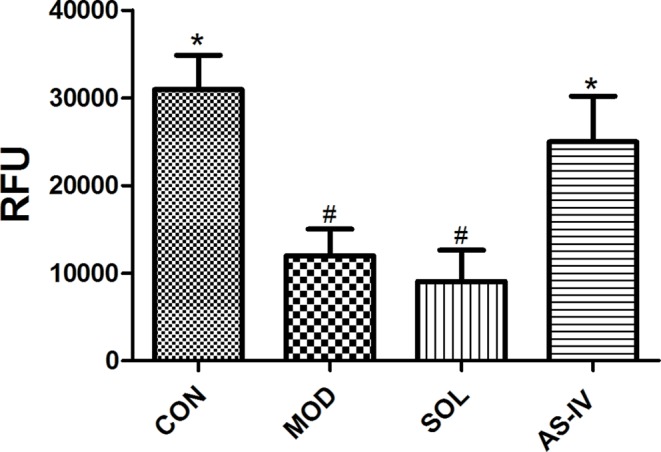
AS-IV prevented ethanol-induced disruption of mitochondrial membrane potential. Significant difference (*P < 0.05) when compared with the model group. Significant difference (#P < 0.05) when compared with the control group. Significant difference when compared with the solvent group.

The relative fluorescence value was detected by a microplate reader at the ratio of 590 nm/490 nm of fluorescence to evaluate the mitochondrial membrane potential. The results indicated that ethanol treatment decreased the mitochondrial membrane potential in the mucosal cells, pretreatment with AS-IV significantly prevented it (*P* < 0.05) (**Figure 4**).

### The Effect of AS-IV on Translation of Bax and Bcl-2

**Figure 5:**
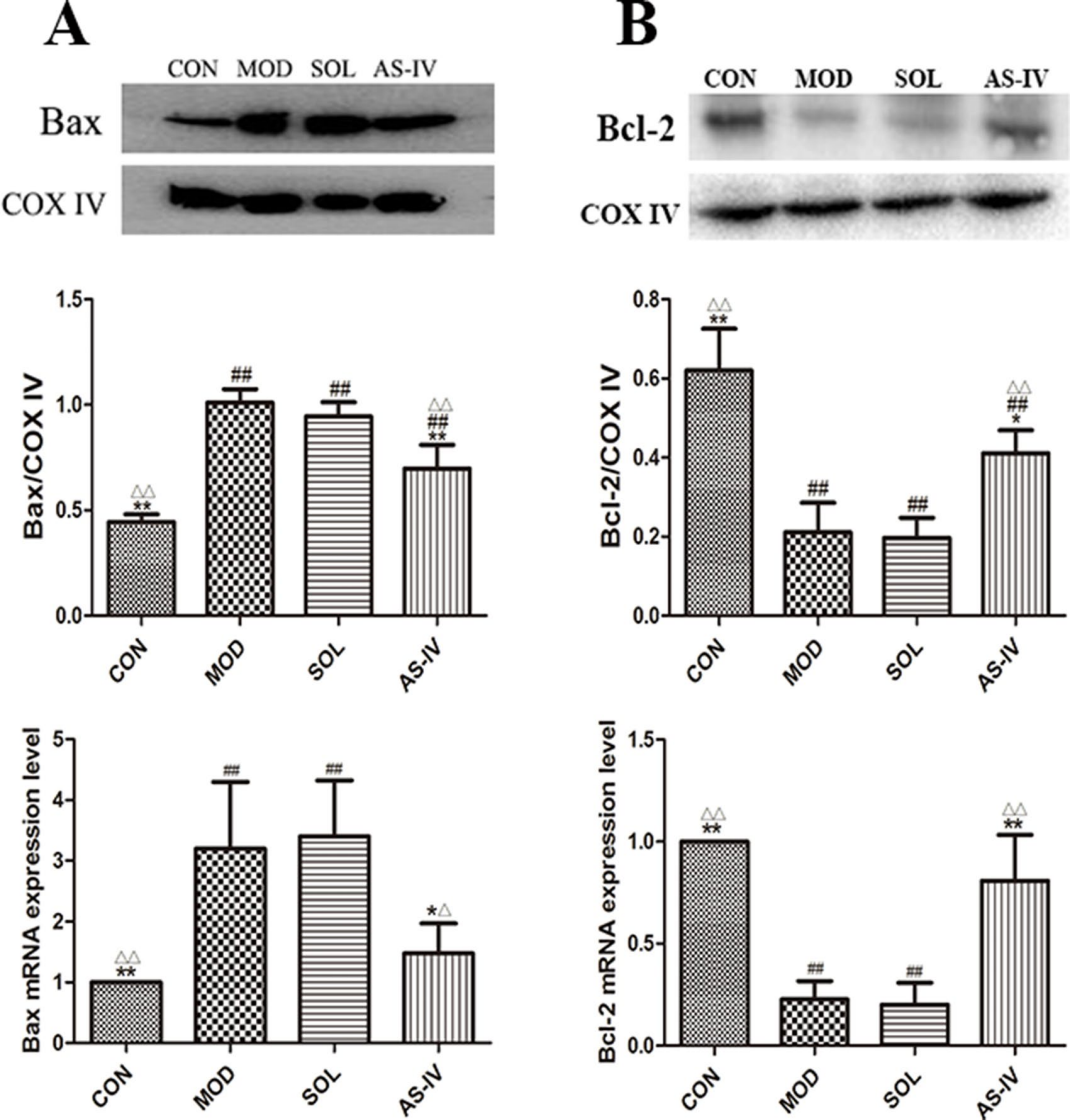
Western blot analysis of Bax and Bcl-2 proteins in mitochondrial fraction and the RT-qPCR analysis of mRNA expression of Bax and Bcl-2. **(A)** The expression of bands and densitometry analysis of Bax in mitochondrial fraction and the expression level of Bax mRNA; **(B)** The expression of bands and densitometry analysis of Bcl-2 in mitochondrial fraction and the expression level of Bcl-2 mRNA. Significant difference (**P < 0.01, *P <0.05) when compared with the model group. Significant difference (##P < 0.01) when compared with the control group. Significant difference (∆∆P < 0.01, ∆P < 0.05) when compared with the solvent group.

Bcl-2 and Bax expressed in mitochondrial membrane are a pair of key proteins that modulate apoptosis of the mitochondrial pathway. The changes in their expression on mitochondrial membrane are associated with the dysfunction of MPTP. After 1 h of ethanol stimulation, the expression of proapoptotic protein Bax in the mitochondria of gastric tissue cells was significantly increased, along with reduction in antiapoptotic protein Bcl-2. AS-IV pretreatment, on the other hand, significantly attenuated ethanol-induced changes of Bax (*P* < 0.01) as well as Bcl-2 (*P* < 0.05) (**Figure 5**).

### The Effect of AS-IV on Cytochrome C and AIF

**Figure 6:**
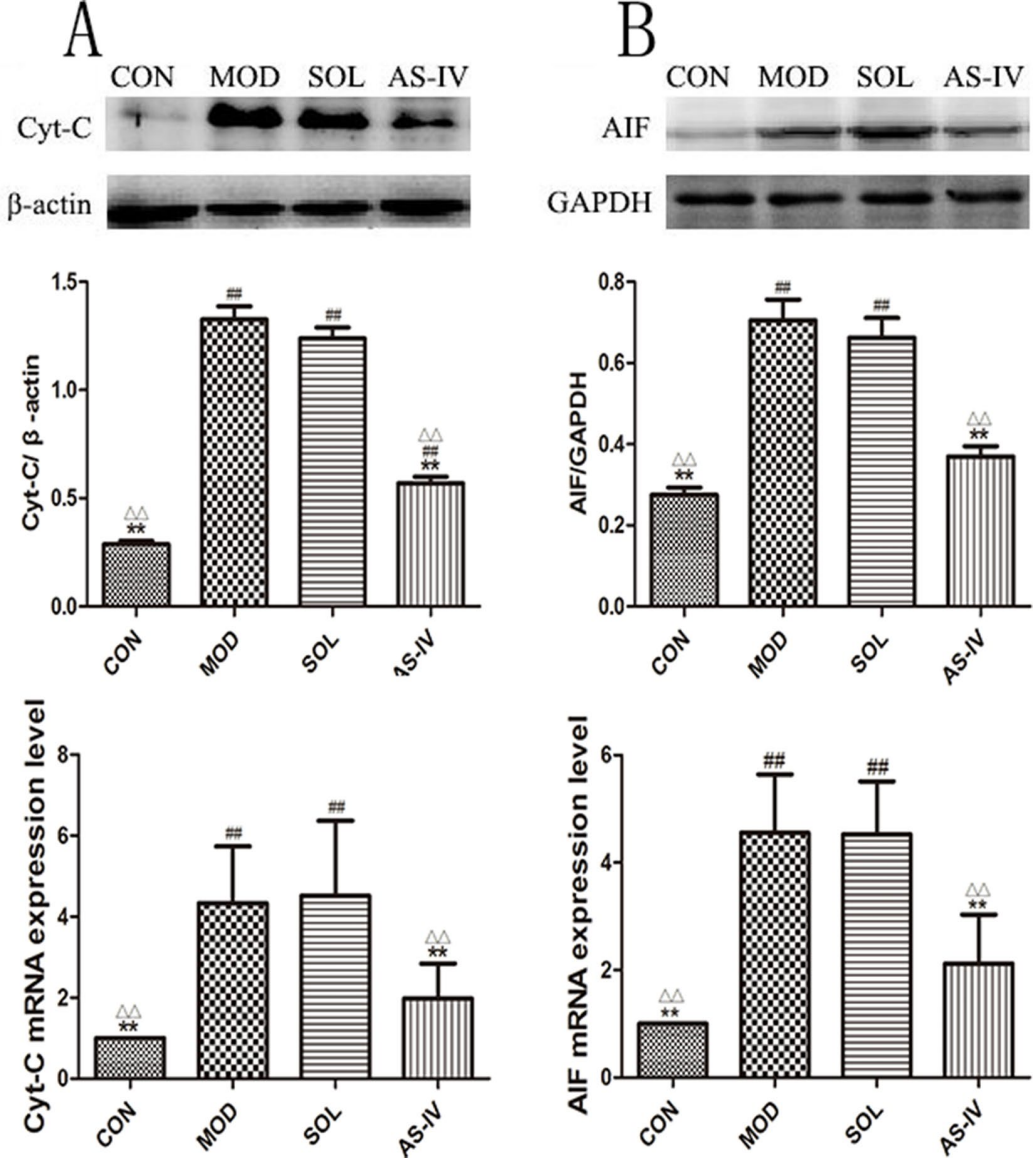
AS-IV prevented the release of Cyt-c and AIF to block ethanol-induced gastric mucosal cell apoptosis. **(A)** The expression of bands and the densitometry analysis of Cyt-c in cytosol and the expression level of Cyt-c mRNA; **(B)** The expression of bands and the densitometry analysis of AIF in cytosol and the expression level of AIF mRNA. Significant difference (**P < 0.01) when compared with the model group. Significant difference (##P < 0.01) when compared with the control group. Significant difference (∆P < 0.01) when compared with the solvent group.

Cytochrome c and AIF are mitochondria-associated apoptosis-related proteins which are released to cytosol to activate the caspase-9 and trigger apoptotic cascade when the mitochondrial membranes are dysfunctional. The Western blot results showed that the contents of cytochrome c and AIF in cytosol were significantly increased after ethanol treatment (*P* < 0.01) compared with the control group (**Figure 6**), which indicated the release of cytochrome c and AIF from the mitochondria to the cytosol. Correspondingly, AS-IV pretreatment caused significant block of ethanol-induced release of cytochrome c and AIF to the cytosol (*P* < 0.01). The model group was not different with the solvent group.

### The Effect of AS-IV on Activity of Caspase-3 and Caspase-9

**Figure 7:**
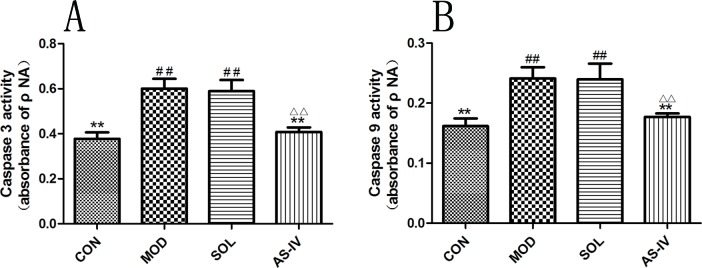
AS-IV inhibited ethanol-induced activation of caspase-9 and caspase-3 in the gastric mucosa. **(A)** Analysis of caspase-3 activity; **(B)** Analysis of caspase-9 activity. Significant difference (**P < 0.01) when compared with the model group. Significant difference (##P < 0.01) when compared with the control group. Significant difference (∆∆P < 0.01) when compared with the solvent group.

Ethanol treatment significantly stimulated the activity of caspase-3 (*P* < 0.01), which was prevented by pretreatment with AS-IV (*P* < 0.01). In the meantime, the caspase-9 was activated as well, and AS-IV performed a negative role in its activation (**Figure 7**). The protein bands and densitometry analysis of Caspase-3 showed in **Table 1**.

### The Effect of AS-IV on Caspase-8 and Bid

**Figure 8:**
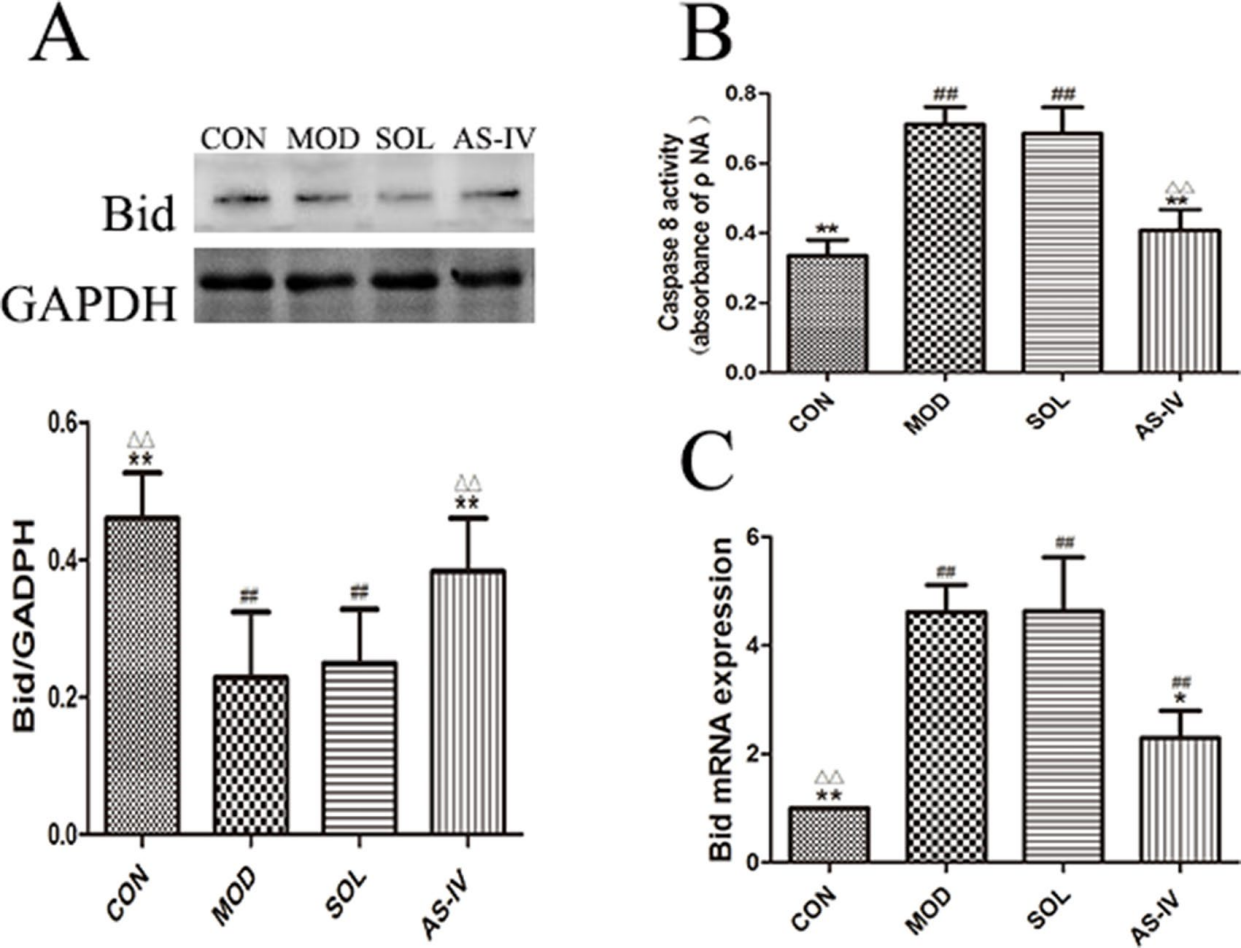
AS-IV inhibited ethanol-induced activation of caspase-8 and mediated expression of pro–apoptotic factor Bid protein in the gastric mucosa. **(A)** The expression of bands of Bid in cytosol and the densitometry analysis; **(B)** Analysis of caspase-8 activity; **(C)** Analysis of the Bid mRNA expression. Significant difference (**P < 0.01, *P < 0.05) when compared with the model group. Significant difference (##P < 0.01) when compared with the control group. Significant difference (∆∆P < 0.01) when compared with the solvent group.

As shown in **Figure 8B**, the activity of caspase-8 in gastric mucosal cells was significantly increased after ethanol treatment (*P* < 0.01), and pretreatment with AS-IV could prevent its change (*P* < 0.01). Meanwhile, the expression of Bid protein in cytosol was decreased after ethanol stimulation (*P* < 0.01) (**Figure 8A**); on the contrary, the mRNA expression of Bid was significantly upregulated (*P* < 0.01) (**Figure 8C**). Pretreatment with AS-IV reversed the reduction of Bid protein and the increase in mRNA (*P* < 0.01 or *P* < 0.05).

### The Effect of AS-IV on mRNA Expression of Apoptotic Factors

The mRNA expressions of cytochrome c, AIF, and Bax were significantly increased accompanied by the decreasing expression of Bcl-2 mRNA in gastric mucosal tissue after ethanol treatment (*P* < 0.01). Pretreatment with AS-IV reversed the changes of mRNA expression of these apoptosis factors (*P* < 0.01 or *P* < 0.05).

## Discussion

The present study is perhaps the first detailed report of the mechanism of ethanol-induced gastric mucosal injury in rat in the aspect of mitochondrial oxidative stress and mitochondria-dependent pathway of apoptosis. It is known that oxidative stress and associated dysfunction in mitochondria, which leads to triggering of the mitochondria-mediated apoptosis, play a crucial role in the development of gastric mucosal injury induced by nonsteroid anti-inflammatory drugs (NSAIDs), *Helicobacter pylori*, and stress. Our results indicated that mitochondrial oxidative stress and mitochondria-dependent pathway of apoptosis also are involved in the ethanol-induced gastric mucosal injury. AS-IV presents the gastroprotective effect by scavenging mitochondrial lipid peroxide and preventing gastric mucosal apoptosis through inhibition of the mitochondrial pathway.

Mitochondrion is involved in many necessary processes for cell survival, such as redox reactions and balance of calcium ion, and acts as the main intracellular source of energy and ROS. Oxidative-stress-mediated structural and functional alteration of mitochondrion caused by the augmentation of ROS generation is found to be central to the pathogenesis of many degenerative diseases (Laine and Weinstein, 1988; Smith et al., 2003; Ishii et al., 2011). Development of mitochondrial oxidative stress in the rat gastric mucosa during NSAIDs (Maity et al., 2009) and *Helicobacter pylori* (Calvino-Fernandez et al., 2008) treatments were reported earlier. The results of this study show that significant mitochondrial oxidative stress revealed in gastric mucosa of rats administrated with ethanol characterized by accumulation of MDA and consumption of GSH and Mn-SOD in mitochondria. The antioxidative stress action of AS-IV was reflected in these processes by inhibition of consumption of GSH along with reduction in MDA. As a SOD isoform only expressed in mitochondria, Mn-SOD activity presented increasing trend in AS-IV group in this study. However, the difference between the model and AS-IV groups was not significant, which indicates that AS-IV inhibits mitochondrial oxidative stress mainly by inhibiting lipid peroxidation, and its scavenging effect on superoxide anion radical in mitochondria was limited.

In contrast to necrosis, apoptosis is associated with profound structural changes and biochemical events, leading to irreversible cell destruction including blebbing, cell shrinkage, nuclear fragmentation, chromatin condensation, and chromosomal DNA fragmentation, which could be detected by TUNEL. The results of TUNEL assay of this study indicated that pretreatment with AS-IV alleviated the apoptosis of gastric mucosal cells, which was induced by ethanol remarkably. As one of the major intrinsic pathways of apoptosis, mitochondrion-related apoptotic pathway could be initiated by mitochondrial oxidative stress and featured by the upregulation of Bcl-2 family of proapoptotic proteins Bax, Bak, and/or downregulation of antiapoptotic Bcl-2 and BclxL (Cory and Adams, 2002). The relative ratio of pro- to antiapoptotic proteins modulates the opening of mitochondrial permeability transition pore (MPTP), which further leads to a decrease in mitochondrial membrane potential (Galluzzi et al., 2007). Furthermore, the opening of MPTP results in the release of apoptosis-promoting factors including Cyt-c, apoptosis-inducing factor (AIF), and endonuclease G that exist in intermembrane space of the mitochondria. Cyt-c in cytosol forms a complex with apoptotic protease activating factor 1 (Apaf-1) and procaspase-9, inducing the activation of the caspase-3, which brings about alterations of apoptosis. AIF is a caspase-independent death effector that can allow nuclei to undergo apoptotic changes independently (Bano and Prehn, 2018). Ethanol treatment in this study was found to decrease mitochondrial membrane potential of the gastric mucosal cells. On the other hand, our results revealed that, after ethanol administration, the amount of Cyt-c and AIF released from the mitochondria into the cytoplasm was significantly increased, and this was accompanied with increased expression of Bax and reduced expression of the Bcl-2 protein in mitochondria. Correspondingly, the activity of caspase-3 was increased, and gastric mucosal cells displayed apoptotic damage. These are evidence that the activation of mitochondrial pathway apoptosis is involved in the development of ethanol-induced gastric mucosal injury. AS-IV blocks the mitochondrial pathway of apoptosis through the inhibition of Bax expression and stimulation of Bcl-2 expression, thereby inhibiting Bax translocation to mitochondria and subsequent collapse of mitochondrial membrane potential, as well as Cyt-c and AIF release.

As an initiator and apical activator caspase, caspase-8 plays a central role in apoptosis. It is induced by Fas and various apoptotic stimuli like TNF-α and IL-1βin process of cell death (Tummers and Green, 2017). The active caspase-8 not only activates downstream executioner caspase-3 straightway in death receptor pathway but also converge to the mitochondrial pathway through cleaving Bid that is a member of Bcl-2 protein family. The Bid peptide segment tBID at the outer mitochondrial membrane binds with Bax to induce Cyt-c release through a functional translocase of the outer membrane complex (Kantari and Walczak, 2011). Our recent study indicated significant upregulation of inflammatory cytokines such as TNF-α and IL-1β in gastric mucosal cells during ethanol-induced gastric mucosal damage (Qin et al., 2017). Therefore, we further examine the activity of caspase-8 and expression of Bid in cytoplasm in this study to ascertain whether the death signaling from different pathways merges with the mitochondrial pathway. Interestingly, our data show that, after ethanol administration, the activity of caspase-8 and caspase-3 were significantly increased, along with Bid protein downregulation and Bid mRNA upregulation, whereas AS-IV pretreatment could significantly reverse these changes. As for the inconsistent expression of Bid protein and mRNA, it is considered that the 22-kd Bid protein in the cytoplasm was cleaved by the active caspase-8 into a 15-kd tBid within 1 h of alcohol treatment, when the new protein has not yet been synthesized. In the meantime, the tBid bound with Bax at the outer mitochondrial membrane and participated in subsequent cell death pathway. Thus, we detected a decrease in the expression of Bid in the cytoplasm. However, due to the stimulation of apoptosis signals, DNA of apoptosis-related factors in the nucleus had begun to transcribe the mRNA to further promote the process of apoptosis, resulting in the increasing expression of Bid mRNA. It is indicated that, in the process of ethanol-induced gastric mucosal injury, the apoptotic signal of death receptor pathway has merged with the mitochondrial pathway through caspase-8 and Bid. The AS-IV, in this study, suppresses the ethanol-induced gastric mucosal cell apoptosis by not only modulating the expression of mitochondrial protein Bax/Bcl-2 but also inhibiting the activation of caspase-8 and breakage of Bid in cytoplasm.

AS-IV is an insoluble triterpenoid saponins which is often prepared into suspension for gastric administration *in vivo* experiments. However, it is all known that oral administration is inferior to intraperitoneal injection in an experiment of protection of gastric mucosal injury because of the vestigial of drug and solvent in the stomach may affect the model prediction effect. Therefore, a patent organic solvent was used in this study to prepare AS-IV injection referred to our previous studies (Sun Xue-lian, 2011; Qin et al., 2017). Same results as in previous studies, the solvent did not affect the result of the experiments in the present study, where the solvent group was no different with the model group.

In conclusion, this present study documents that ethanol augments intramitochondrial ROS generation and develops mitochondrial oxidative stress which induces the apoptosis of gastric mucosal cell through mitochondria-dependent pathway. In addition to the inhibition of inflammation, AS-IV also protects the gastric mucosal injury from ethanol by not only blocking mitochondrial oxidative stress but also preventing mitochondria-mediated apoptosis.

## Data Availability

All datasets generated for this study are included in the manuscript and the Supplementary files.

## Ethics Statement

This study was carried out in accordance with the principles of China Regulations on the Administration of Laboratory Animals, the Decree No. 2 of National Science and Technology Commission of the People’s Republic of China. The protocol was approved by the animal ethics committee of the Guangzhou University of Chinese Medicine, China.

## Author Contributions

SQ and JY contributed equally to this work. KH, SQ, and JL conceived and designed the experiments. SQ, JY, ZF, YZ, and JL performed the experiments. SQ and SH analyzed the data. SQ and JY checked the drafts and finalized the manuscript.

## Funding

This work was financially supported by Special Fund for Public Welfare Research Institutes of Fujian Science and Technology Department (No. 2016R1033-1), Special Research Program of Translational Medicine of Traditional Chinese Medicine of Guangdong Provincial Hospital of Chinese Medicine (No. YN2015MS08), and Scientific Research Project of Guangdong Administration of Traditional Chinese Medicine (No. 20192024).

## Conflict of Interest Statement

The authors declare that the research was conducted in the absence of any commercial or financial relationships that could be construed as a potential conflict of interest.
